# The effectiveness of liver transplantation in reducing lipid levels in Saudi children with homozygous familial hypercholesterolemia

**DOI:** 10.3389/fcvm.2024.1454638

**Published:** 2024-11-15

**Authors:** Abdullah Al-Ashwal, Salman Al-Mansour, Mohammed Al-Shagrani, Talal Al-Gofi, Dieter Broering, Raghad Alhuthil

**Affiliations:** ^1^ Department of Pediatrics, King Faisal Specialist Hospital and Research Centre, Riyadh, Saudi Arabia; ^2^ Department of Pediatrics, College of Medicine, Qassim University, Buraydah, Saudi Arabia

**Keywords:** homozygous, familial hypercholesterolemia, cholesterol, LDL-C, liver transplant

## Abstract

**Introduction:**

The lipid profiles of patients aged <15 years who have been diagnosed with homozygous familial hypercholesterolemia (HoFH) at King Faisal Specialist Hospital & Research Center (Riyadh) were examined.

**Methods:**

The total cholesterol and low-density lipoprotein cholesterol (LDL-C) levels of 17 patients were measured on initial presentation and compared with the levels measured after pharmacological treatment and then again after liver transplantation.

**Results:**

At the end of the pharmacological treatment, the total cholesterol levels decreased by an average of 3.79 mmol/L (reduced by 15.40%) (*P* < 0.001), and LDL-C levels decreased on average by 2.73 mmol/L (reduced by 13.46%) (*P* = 0.014). However, in two patients, LDL-C levels increased by 5.42% and 9.03% after pharmacological treatment. Conversely, the lipid values measured after liver transplantation decreased significantly nearly to within normal and borderline limits. The post-transplant total cholesterol and LDL-C levels declined by a mean of 19.96 mmol/L (reduced by 81.04%) and 17.47 mmol/L (reduced by 84.27%), respectively (*P* < 0.001 for both).

**Discussion:**

These findings suggest that liver transplantation provides a more effective means to reduce elevated total cholesterol and LDL-C levels in patients with HoFH. Although liver transplantation is considered a better treatment for FHoH, risks, complications, and donor organ shortage may present problems.

## 1 Introduction

Pediatric patients with homozygous familial hypercholesterolemia (HoFH) present with signs and symptoms at a very early age. They suffer from this disorder from birth and have unusually high lipid levels, which are not discovered early unless an index case is identified or incidentally found in the blood test. HoFH usually results from autosomal-dominant inheritance of genetic aberrations in the coding region for low-density lipoprotein receptors (LDLRs) (1). Similar inherited disorders that may affect pediatric patients can produce similar phenotypes having mutations in the genetic material for apolipoprotein B100 (apoB100), proprotein convertase subtilisin/kexin type 9 (PCSK9), and autosomal recessive hypercholesterolemia due to LDLRAP1 mutations (1, 2).

HoFH results from the same mutation in both LDLR alleles, whereas the heterozygous form only has a mutation in one allele. Individuals with different mutations in both LDLR alleles are considered compound heterozygotes (1, 2). These children will require early interventions from a young age to control and manage their cholesterol and low-density lipoprotein cholesterol (LDL-C) levels before worsening conditions develop.

The incidence of atherosclerosis in children has increased over the last two decades, particularly in the Middle East because of changes in lifestyle, dietary habits, and environmental and genetic factors. In the Middle East, the exact prevalence of HoFH is unknown; however, it may be higher than in other parts of the world because of the high incidence of consanguineous marriages (3). According to global estimates for HoFH, this disease may affect 1 in 1 million individuals; however, the prevalence of this lipid abnormality varies from one country to another (3). The negative effect of this disease could be more serious for pediatric patients in the Middle East because some treatments may be unavailable and access barriers in some countries.

In 2001, El-Hazmi and Warsy examined lipid abnormalities in 2,914 Saudi children aged 1–15 years (4). The participants were randomly selected from a national household screening program and divided into 14 groups according to age. The cholesterol and triglyceride levels of the children were measured. The participant’s cholesterol results were evaluated using Kuiterovich’s reference ranges, that is, the desirable, borderline, and high-risk cholesterol levels were <4.36, 4.36–4.88, and ≥5.13 mmol/L, respectively. The desirable, borderline, and high-risk triglyceride levels were <0.825, 0.825–1.089, and ≥1.1 mmol/L, respectively. The cholesterol findings from this study indicated that 7.72% and 1.55% of Saudi children had borderline and high-risk levels, respectively. Regarding triglycerides, 1.4% and 0.55% of Saudi children had borderline and high-risk levels, respectively (4). These findings suggest sufficient cholesterol and triglyceride monitoring in Saudi children. Dietary factors such as the consumption of low-fat food and more vegetables can provide some benefits for children (5).

The criteria for the diagnosis of HoFH are usually based on a combination of several clinical observations: the initial presentation may be a child with skin lesions, LDL-C level of 650–1,000 mg/dl (6), and serum fasting cholesterol level of >13 mmol/L (>500 mg/dl) (7, 8), and positive mutation in LDLR, apoB100, PCSK9, or LARD gene.

Patients usually exhibit the physical signs and symptoms of the disease, such as xanthomas, corneal arcus, and/or atherosclerosis at an early age (within the first decade of life) (8). Children with the homozygous form usually present with severe symptoms and are seriously ill at a young age. Without proper treatment to lower plasma cholesterol levels before the development of coronary artery disease, these children have a poor prognosis.

Many modalities have been used for the treatment of patients with familial hypercholesterolemia including diet, drugs, plasmapheresis, and portocaval shunts (9). These therapeutic regimens have shown limited effectiveness in reducing the total cholesterol and LDL-C levels in the plasma (9). Additionally, particularly for patients with HoFH, these treatments only provide temporary solutions for patients with hypercholesterolemia who may still experience symptoms of atherosclerotic cardiovascular disease (9).

Plasma lipoprotein apheresis (LDL apheresis) provides an option in combination with medical therapy, which has been shown to reduce circulating LDL-C levels by up to ≥60% (9). Treatment using plasma lipoprotein apheresis may be difficult because it may only be offered at a few facilities, is expensive, and may not be available for certain ages. This procedure must be performed weekly week or biweekly, which requires a significant amount of time regularly (1–3, 7, 8).

Portocaval shunt surgery has demonstrated limited success in reported cases where the LDL-C level was reduced by ≥25% in approximately 80% of the patients (10). This treatment method intends to decrease the production rates of LDL-C and apoB100. Portocaval shunts have limited capacity to increase the fractional rate of clearance of these lipids; therefore, plasma lipid concentrations remain high (10).

A previous study demonstrated that liver transplantation (LT) was a highly effective means to lower the LDL-C level up to ≥80% in recipients (9–11). The transplanted liver provides normal LDLR activity, which leads to a decrease in the production rates of LDL-C and apoB100 while increasing the fractional catabolic rate (9–11). Because patients with HoFH were also found to be more responsive to medication therapy after LT, LT was considered a better treatment than portocaval shunt surgery for these patients (10). Thus, this study aimed to compare the effectiveness of LT with drug therapy in reducing total cholesterol and LDL-C levels. The findings could have significant value for Saudi children suffering from HoFH who are not able to benefit from other treatments.

## 2 Methods

A retrospective study was conducted to review the treatment outcomes of 17 patients diagnosed with HoFH who had undergone LT between 2018 and 2019.

The study included pediatric patients aged 14 years and younger who were diagnosed with HoFH and followed at the Pediatric Endocrine Clinic at King Faisal Specialist Hospital in Riyadh, Saudi Arabia. Patients who did not undergo LT were excluded.

The pharmacological treatment consisted of Ezetimibe initiated as early as 4 years (dose: 10 mg once per day orally), followed by Atorvastatin (Lipitor) after the age of 6 years (dose: 10–40 mg once per day orally depending on patient age and weight).

The levels of total cholesterol (reference: normal <5.2 mmol/L; borderline high 5.2–6.2 mmol/L; high ≥6.2 mmol/L) and LDL-C (normal <2.59 mmol/L; low risk 2.59–3.34 mmol/L; borderline high 3.37–4.12 mmol/L; high 4.14–4.90 mmol/L; very high ≥4.92 mmol/L) were measured before and after drug therapy. Subsequently, all 17 patients had undergone LT. Similarly, total cholesterol and LDL-C levels were evaluated before and after LT.

Statistical data analysis was performed using STATA version 18 to examine the effectiveness of each treatment modality on lipid levels using the paired *t*-test due to normality assumption by the Shapiro–Wilk test. A *p*-value of <0.05 was considered significant. Figures were generated using Microsoft Excel 2016.

This study was cleared and approved by the Ethics Committee at King Faisal Specialist Hospital and Research Center (No. 2245271). A waiver of consent was granted from the ethics committee, given the retrospective nature of the study.

## 3 Results

A total of 17 patients were included in the study, comprising 9 males (52.94%) and 8 females (47.06%), aged between 10 months and 3 years. A positive family history of HoFH and consanguinity were reported in 100% and 94.12% of the cases, respectively (Table 1).

**Table 1:** Summary of study participants (n = 17).

Family ID	Sex	Age at presentation	Symptoms	Mutation	Family history	Consanguinity	Echo findings	Apheresis	Medications	Follow-up period (years)
1A	M	1 year	Xanthomas	c.2027 delG; p.Gly6 in *LDLR*	Positive	1st cousin	Normal	No	None	10
1B	F	2 years and 7 months	Xanthomas	c.2027 delG; p.Gly6 in *LDLR*	Positive	1st cousin	Thickened Aortic root	No	Ezetrol, simvastatin, Zocor	15
1C	F	1 Year	Xanthomas	c.2027 delG; p.Gly6 in *LDLR*	Positive	1st cousin	Normal	No	Zocor	13
2	F	2 years	Xanthomas	c.2027 delG; p.Gly676fsX 33 in *LDLR*	Positive	Same tribe	Mild supra AS and atherosclerosis in the AA	No	Pravastatin, Ezitimibe, Rosuvastatin	16
3A	F	2 years	Xanthomas, Corneal arcus	c.2027 delG; p.Gly676fsX 33 in *LDLR*	Positive	2nd cousin	Normal	No	Lipostat, Ezitimibe, Zocor	16
3B	F	1 year and 7 months	Xanthomas, Corneal arcus	c.2027 delG; p.Gly676fsX 33 in *LDLR*	Positive	2nd cousin	Normal	No	Parvastatin	16
4	M	2 years	Xanthomas, Abdominal pain	c.2027 delG in *LDLR*	Positive	1st cousin	Normal	No	Cholestyramin	10
5A	F	3 years	Xanthomas	Not done	Positive	1st cousin	Normal	No	Zocor	11
5B	M	10 months	Asymptomatic	Not done	Positive	1st cousin	Not done	No	None	10
6A	F	3 years	Xanthomas	c.1731G > T;p. W577c in *LDLR*	Positive	1st cousin	Mild AS and narrow AA	No	Lipostat, Ezetimibe	19
6B	F	2 years	Xanthomas	c.1731G > T;p. W577c in *LDLR*	Positive	1st cousin	Normal	No	None	11
7	M	18 months	Xanthomas	Not done	Positive	1st cousin	Mild thickening of the aortic valve	Yes	Zocor, Atorvastatin	24
8	M	3 years	Xanthomas	Not done	Positive	2nd cousin	Severe AS	No	Cholystramin, Simvastatin	11
9	M	2 years	Xanthomas	Not done	Positive	1st cousin	Thickened aortic root wall, calcified atheroma in the distal AA	No	Atorvastatin, Zocor, Ezetrol	21
10	M	1 year	Xanthomas, senile arcus	Not done	Positive	1st cousin	Mild aortic regurgitation	No	Atorvastatin, Ezetimibe	17
11	M	3 years	Xanthomas	Not done	Positive	1st cousin	Multiple atheromas at the aortic valve and AA	No	Cholestyramin, Atorvastatin, Ezetimibe	13
12	M	2 years	Xanthomas	Not done	Positive	1st cousin	Minimal thickened aortic valve	No	Ezetrol	8

M, male; F, female; AS, aortic stenosis; AA, Ascending Aorta.

**Figure 1:**
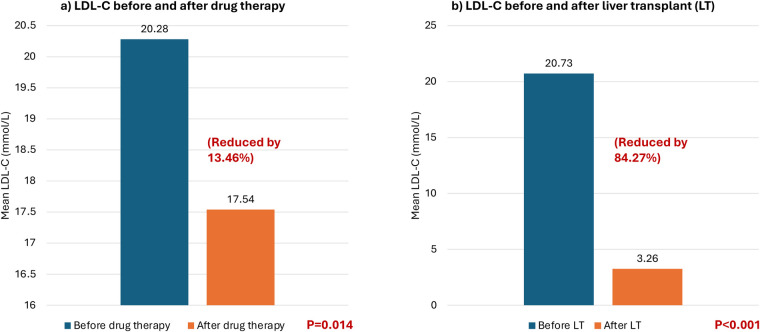
Mean low-density lipoprotein cholesterol (LDL-C) levels (mmol/L) before and after different treatment modalities. **(a)** Comparing LDL-C before and after drug therapy. **(b)** Comparing LDL-C before and after liver transplant.

**Figure 2:**
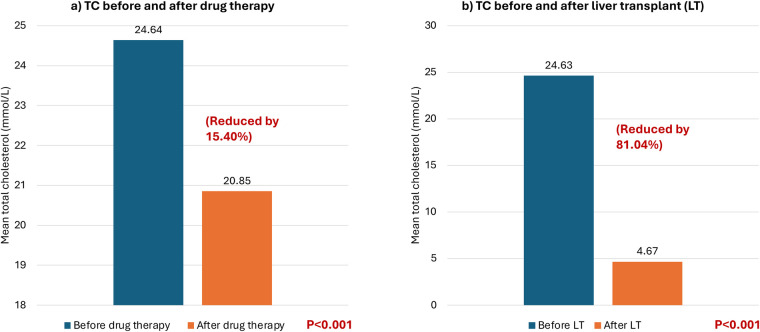
Mean total cholesterol levels (mmol/L) before and after different treatment modalities. Mean total cholesterol levels (TC) (mmol/L) before and after different treatment modalities. **(a)** TC before and after drug therapy. **(b)** TC before and after liver transplant.

At baseline, all patients had total cholesterol levels exceeding 20 mmol/L and LDL-C levels exceeding 16 mmol/L. Thirteen patients were on pharmacological treatment, and their LDL-C levels were examined before and after drug treatment. The patients’ LDL-C levels dropped by an average of 2.73 mmol/L with an SD of 3.45 after pharmacological treatment, except for 2 patients whose LDL-C levels increased by 5.42% and 9.03%, respectively. Overall, the LDL-C levels decreased by 13.46% after pharmacological treatment (*P* = 0.014) (see Table 2 and Figure 1a). Furthermore, the patients' total cholesterol levels decreased by an average of 3.79 mmol/L (reduced by 15.40%) (*p* < 0.001) after pharmacological treatment (Figure 2a).

**Table 2:** Laboratory investigations.

Family ID	TC level at baseline (category)^a^	TC level after medical ttt^b^	TC level after LT (category)^a^	LDL-C level at baseline	LDL-C level after medical ttt^b^	LDL-C level after LT (category)^c^	LT Source	LT Total or Partial	Current Immuno-suppressants	LT complication
1A	25.4 (H)	–	5.59 (B)	21.80 (VH)	–	4.15 (H)	Cadaveric	Total	Tacrolimus 1.1 mg PO BID	None
1B	21.8 (H)	18.6 (H)	3.72 (N)	18.1 (VH)	15.6 (VH)	2.35 (N)	Cadaveric	Total	Tacrolimus 0.5 mg (1 ml) BID, CellCept 3 ml BID	None
1C	21.1 (H)	21.4 (H)	3.70 (N)	16.6 (VH)	17.5 (VH) (increased by 5.42%)	2.30 (N)	Cadaveric	Total	Tacrolimus 0.9 mg PO q12hr	ACR
2	24.4 (H)	20.3 (H)	3.55 (N)	>16.6 (VH)	>16.6 (VH)	2.24 (N)	Cadaveric	Total	Sirolimus 2 mg every other day, Tacrolimus 1 mg BID, Prednisolone 5 mg OD	None
3A	20.7 (H)	19.2 (H)	3.98 (N)	16.9 (VH)	15.8 (VH)	2.95 (L)	Cadaveric	Partial	Tacrolimus 2 mg PO q12hr	None
3B	25.6 (H)	22.2 (H)	4.61 (N)	19.4 (VH)	18.6 (VH)	3.46 (B)	Cadaveric	Total	Tacrolimus 2 mg PO q12hr	Biliary stricture, EBV infection
4	21 (H)	–	4.98 (N)	17.4 (VH)	–	3.85 (B)	Cadaveric	Total	Tacrolimus 0.9 mg PO q12hr	None
5 A	29.9 (H)	23.8 (H)	3.74 (N)	27.8 (VH)	20.55 (VH)	1.85 (N)	Cadaveric	Partial	Tacrolimus 1 mg PO q12hr	Liver graft was infected with HBV and had ACR
5B	25.09 (H)	–	4.74 (N)	23.8 (VH)	–	3.83 (B)	Cadaveric	Total	Tacrolimus 1 mg PO q12hr	None
6A	26.1 (H)	20.6 (H)	6.70 (H)	20.9 (VH)	18.2 (VH)	5.46 (VH)	Cadaveric	Total	Tacrolimus 1.5 mg PO q12hr	None
6B	26.8 (H)	–	5.72 (B)	25.9 (VH)	–	3.58 (B)	Cadaveric	Total	Tacrolimus 1.5 mg PO q12hr	Mild to moderate rejection
7	22.4 (H)	14.4 (H)	4.99 (N)	21.5 (VH)	10.4 (VH)	3.84 (B)	Cadaveric	Total	Tacrolimus 1 mg PO q12hr	None
8	26.09 (H)	22.85 (H)	4.37 (N)	23.86 (VH)	19.63 (VH)	2.49 (N)	Related	Partial	Tacrolimus 0.45 mg PO q12hr	None
9	26 (H)	20.9 (H)	4.12 (N)	>16.6 (VH)	18.1 (VH) (increased by 9.03%)	2.86 (L)	Cadaveric	Partial	Tacrolimus 1.5 mg PO q12hr	None
10	26 (H)	23.7 (H)	4.2 (N)	21.1 (VH)	19.74 (VH)	2.70 (L)	Cadaveric	Partial	Tacrolimus 2 mg BID	None
11	25.1 (H)	20.1 (H)	5.36 (B)	21.5 (VH)	17 (VH)	3.82 (B)	Related	Partial	Switched from Tacrolimus to Cyclosporine 100 mg PO q12hr (doctor decision)	None
12	25.2 (H)	23 (H)	4.89 (N)	22.7 (VH)	20.36 (VH)	3.54 (B)	Living unrelated	Partial	Tacrolimus 1.5 mg BID, CellCept discontinued due to diarrhea	ACR

TC, total cholesterol; ttt, treatment; LT, liver transplantation; LDL-C, low-density lipoprotein cholesterol; H, high; N, normal; B, borderline; VH, very high; L, low risk; ACR, acute cellular rejection; EBV, Epstein–Barr virus; HBV, hepatitis B virus; AA, ascending aorta; AS, aortic stenosis.

All measurements by mmol/L.

^a^ The reference range of total cholesterol (normal <5.2 mmol/L; borderline high 5.2–6.2 mmol/L; high ≥6.2 mmol/L).

^b^ 13 patients only were on pharmacological treatment.

^c^ The reference range of LDL-C (normal <2.59 mmol/L; low risk 2.59–3.34 mmol/L; borderline high 3.37–4.12 mmol/L; high 4.14–4.90 mmol/L; very high ≥4.92 mmol/L).

All seventeen patients subsequently received liver transplantation which caused a significant decrease by 81.04% in total cholesterol in all patients with a mean decrease of 19.96 mmol/L (pre-transplantation: 24.63 mmol/L vs. post-transplantation: 4.67 mmol/L) (*p* < 0.001) (see Figure 2b). Additionally, the LDL-C decreased in all patients by approximately 84.27% with an average reduction of 17.47 mmol/L (pre-transplantation: 20.73 mmol/L (very high reference: ≥4.92 mmol/L) vs. post-transplantation: 3.26 mmol/L (low risk 2.59–3.34 mmol/L) (*p* < 0.001) (see Figure 1b).

Thus, the post-LT total cholesterol level declined to within normal limits (<5.2 mmol/L) in most patients (76.47%) (13/17). While the post-LT LDL-C decreased to within normal to borderline limits (≤4.12 mmol/L) in the majority of cases (88.23%) (15/17) (see Table 2).

All our patients are alive and attending regular follow-up visits at the liver transplantation outpatient clinic with an average follow-up period of 14.2 years and SD of 4.4. Their families are satisfied with their overall progress.

## 4 Discussion

This study revealed the efficacy of LT in reducing total cholesterol and LDL-C levels in patients with HoFH. Previous studies have reported similar cases of HoFH displaying remarkable results (12, 13). Because approximately 70% of damaged LDLRs are located in the liver, LT presents an effective means to replace the defective LDLRs with normally functioning ones (12, 14). LT helped restore the total cholesterol and LDL-C levels to within or near normal ranges in the majority of patients.

These patients with HoFH all had high plasma levels of LDL-C because the LDLRs were unable to function normally in the removal of LDL particles, and biochemically, apoB could have caused atherosclerosis (14, 15). Atherosclerosis in young patients with HoFH may cause such severe coronary artery disease and valvular disease, so they may not have survived much longer than their second decade of life (15).

When comparing post-LT lipid levels to those of a healthy pediatric population, it’s important to note that healthy children typically maintain normal total cholesterol and LDL-C levels (4). In contrast, pediatric patients with HoFH exhibit much higher baseline levels due to a genetic inability to process cholesterol properly (1). Although the post-LT lipid levels in our study significantly improved, may still be slightly higher than those in a healthy pediatric population. This difference can be attributed to the fact that while LT addresses the primary metabolic defect, other factors such as pre-existing vascular damage and the body’s adaptation to the transplanted liver may influence lipid levels (16).

A similar study by Page et al. investigated LT outcomes in 9 HoFH patients with ages ranging from 3 to 26 years. The baseline LDL-C levels were 23 ± 4.1 mmol/L without treatment. Under medical therapy, including maximal statin use, ezetimibe, and LDL-apheresis (LA) in some patients, the mean LDL-cholesterol decreased to 11 ± 5.7 mmol/L (*p* < 0.001). Post-LT, the mean LDL-cholesterol further dropped to 2.6 ± 0.9 mmol/L (*p* = 0.004), with three patients remaining on statins and none on LA. One patient died from acute myocardial infarction three years post-LT, and two others required aortic valve replacements over ten years later. The remaining six patients remained asymptomatic after 8–21 years of follow-up. Thus, LT for HoFH patients was very effective in reducing LDL-C levels in all cases (17).

Several factors may influence the degree of success of LT as a treatment for HoFH. The size of the graft transplanted represents the number of normal receptors available for the recipient, and the cholesterol level of the donor may be a contributing factor to treatment success (18). Although slightly variable results may occur under different circumstances, for most recipients with HoFH, the total cholesterol and LDL-C levels will decline to near-normal levels (19). The risk of complications and side effects does exist for LT patients. Under optimal circumstances, if suitable donor organs are available for transplantation, pediatric patients would undergo LT before acquiring cardiovascular disease (20).

In cases where donor organs are unavailable, family members such as a parent may meet the criteria for living-related LT (20). Living relatives may provide a viable option for pediatric patients in need of LT when a shortage of organ donors exists. Other issues for LT patients could involve the risk of organ rejection and/or side effects of immunosuppressive drug therapy (21). Patients with HoFH have been shown to qualify as donors for Domino LT for other recipients in need of liver grafts (22).

The effect of drug therapy on the total cholesterol and LDL-C levels was examined in this study. Drug therapy reduced the levels of both total cholesterol and LDL-C in Saudi pediatric patients before LT. It lowered the levels of circulating total cholesterol in 12 of 13 patients and LDL-C in 11 of 13 patients only by 10%–15%. A previous study indicated the limited success of medications such as statins, bile acid sequestrants, and fibrates (21). Evinacumab was considered for our patients, but it was approved by the FDA “Food and Drug Administration” later in 2021 to be used for children aged 5 years and above with HoFH (23). Furthermore, newer pharmacological treatment regimens are being explored, such as antisense oligonucleotides to apoB that restrict mRNAs (mipomersen), and a molecule that blocks apoB production via the microsomal triglyceride transfer protein mechanism of action (lomitapide) (21). While lomitapide remains experimental for pediatric use, a recent phase 3 trial demonstrated its potential by achieving significant and clinically meaningful LDL cholesterol reduction. These findings suggest that lomitapide could serve as an effective, LDL receptor-independent treatment option for pediatric patients with HoFH (24).

In the present study, the drug therapy was observed much less effective than LT in lowering plasma lipid levels. However, this is a retrospective single-center study; more advanced investigations are needed to develop improved pharmacological treatments for patients with HoFH.

## 5 Conclusion

The drug therapy had a much smaller reduction effect on both total cholesterol and LDL-C levels. Although it lowered the total cholesterol level on average more than the LDL-C level (3.79 vs. 2.73 mmol/L), the overall benefit of drug therapy was much less than that of LT. Additionally, the lipid levels in two patients increased after the initiation of drug therapy. Although the findings from the drug therapy analysis suggest a greater effect on total cholesterol overall, patients’ total cholesterol levels after treatment were still extremely high. Similarly, for LDL-C, the LDL-C levels after drug therapy continued to be unacceptably high in all 17 Saudi patients.

The observed decline in post-LT lipid levels to near-normal limits in HoFH patients is a significant clinical improvement and brings them closer to the lipid profiles seen in healthy children. These findings support LT as the superior method of treatment for young Saudi patients. However, the residual elevation in some cases reflects the ongoing need for careful monitoring and potentially adjunctive lipid-lowering therapies to achieve optimal cardiovascular outcomes.

Additionally, the risks of LT cannot be overlooked because complications such as organ rejection, infections, and immunosuppressive drugs could affect patient outcomes. Thus, the benefits of LT must be weighed against the risks for young patients. The risk of cardiovascular disease must also be assessed for each patient, and early interventions may be necessary before the onset of atherosclerosis symptoms.

## Data Availability

The raw data supporting the conclusions of this article will be made available by the authors, without undue reservation.
